# Does early specialization provide an advantage in physical fitness development in youth basketball?

**DOI:** 10.3389/fspor.2022.1042494

**Published:** 2023-01-10

**Authors:** André A L Soares, Ahlan B Lima, Caio G Miguel, Luciano G Galvão, Thiago J Leonardi, Roberto R Paes, Carlos E Gonçalves, Humberto M Carvalho

**Affiliations:** ^1^ Department of Physical Education, School of Sports, Federal University of Santa Catarina, Florianópolis, Santa Catarina, Brazil; ^2^ School of Physical Education, Physiotherapy and Dance, Federal University of Rio Grande do Sul, Porto Alegre, Rio Grande do Sul, Brazil; ^3^ Faculty of Physical Education, University of Campinas, Campinas, São Paulo, Brazil; ^4^ Faculty of Sport Sciences and Physical Education, University of Coimbra, Coimbra, Portugal

**Keywords:** youth sports [MeSH], Bayesian methods, statistics, young athletes, biological maturation, selection

## Abstract

The present study examined the influence of the specialization onset on the magnitude and patterns of changes in basketball-specific physical fitness within a competitive season and developmental fitness trends between 11 and 17 years in young basketball players. Repeated measures of 181 young basketball players (female, *n* = 40; male, *n* = 141) were examined. Anthropometry, age, estimated maturity status, and basketball-specific physical fitness (assessed with the countermovement jump, line drill, and yo-yo intermittent recovery level-1 and fitness score) were considered. Players were grouped by the onset of specialization as related to biological maturation milestones (pre-puberty, mid-puberty, and late-puberty specialization). The within-season and developmental changes in physical fitness were fitted using multilevel modeling in a fully Bayesian framework. The fitness outcomes were similar between-player and within-player changes when grouped by specialization across a season. Fitness improvements across a season were apparent for female players, while male players maintained their performance levels. There was no variation in the patterns of physical fitness development between 11 and 17 years associated with the onset of specialization. Conditional on our data and models, the assumption that early sport specialization provides a physical fitness advantage for future athletic success does not hold.

## 1 Introduction

The notion that early sport specialization is essential for performance development and attainment of expertise is deeply entrenched in youth sports (1). The notion has been based on the deliberate practice theory (2) applied to sports (3–5). An underlying premise of the theory applied to sports is that highly specific training with appropriate supervision at an early age will improve the functioning of the body's main organ systems beyond what normal growth and development or more diversified physical activities can achieve (6). Unfortunately, clear evidence is virtually nonexistent to address whether there is a physiological advantage of early specialization.

The debate about the merits and risks of specialization in youth sports has recently increased (7–12). Specialization, in general, can be conceptualized as year-round participation in a single “signature” sport, with limited involvement in potential sport alternatives, with a deliberate focus on training and development in the pursuit of elite status (10, 13–15). Youth sports participation and specialization can be conceptualized as a continuum, but there are no clear references for early or late specialization (9, 14). An important caveat remains, given the lack of consensus about the definition of early specialization (9). We argue that specialization can be defined and interpreted relative to pubertal growth (4, 14). Specifically, we can consider the onset of specialization as related to biological maturation milestones that describe the pubertal growth period, i.e., the age of initiation of the pubertal growth spurt and the age at peak height velocity (PHV). Based on growth studies data (16), the biological maturation milestones can be defined using meta-analysis (14). Players can be labeled as follows: pre-puberty specialization, when specialization occurs before the onset of pubertal growth (i.e., early specialization); mid-puberty, when specialization occurs between the onset of pubertal growth and the age of PHV (i.e., during pubertal growth); late-puberty specialization, when specialization occurs after pubertal growth (i.e., after the age of PHV).

In this study, we focus on youth basketball. Coaches and youth basketball programs generally promote engagement and commitment to basketball practice in supervised training contexts as early as five years of age (17). In basketball, body dimensions and specific physical fitness, including vertical jumps, sprints with direction changes, and intermittent endurance, are important determinants of performance at high competitive levels (18). Consequently, decisions of selection/promotion in youth basketball are substantially influenced by players' physical fitness and size. On the other hand, the partition of maturity-associated variation in body size and physiological functions is warranted to interpret appropriately young players' performance (19, 20). However, the increased observations in youth basketball continue to be mostly based on cross-sectional surveys (14, 19, 21–23), despite the persistent call for longitudinal designs.

Coaches generally interpret ﬁtness to be maintained or improved during a season and across adolescence (24). Therefore, understanding the development patterns across a competitive season and adolescence may provide valuable information to coaches and stakeholders to elevate the quality of their training interventions and decision-making, especially at early ages. Unfortunately, data analyzing physical fitness responses across a competitive season and adolescence among young basketball players is limited (20, 24, 25). Furthermore, sexual dimorphism with pubertal growth may complicate the interpretations of the influence of specialization on physical fitness development in youth basketball. Sex differences in timing and tempo of pubertal growth and maturation are substantial (16) and merit consideration when examining the physical fitness development of adolescent basketball players.

We examine the validity of the assumption supporting early specialization, stating that there are basketball-specific physical fitness advantages of early specialization in young players (1, 6). Hence, we examined the influence of the specialization onset on the magnitude and patterns of changes in basketball-specific physical fitness within a competitive season and the developmental trends of fitness from 11 to 17 years in young basketball players. To allow a comprehensive interpretation, we illustrate the use of multilevel modeling in a fully Bayesian framework to estimate the variation in the outcomes accounting for repeated measures and cross-classified nesting, i.e., within players' variation across the season and between player variation in the physical fitness changes responses by the onset of specialization, sex, competitive age group, and estimated maturity status.

## 2 Materials and methods

### 2.1 Participants and study design

This study considered data from surveys with repeated measures collected from competitive seasons from 2015 to 2019 in youth basketball. The sample included 181 youth basketball players (female players, *n* = 40; male players, *n* = 141) aged between 11.7 to 17.0 years at pre-season. Specifically, in this study, we considered repeated measures across a competitive season of players from under-13, under-15, and under-17 teams at pre-season (February/March), mid-season (July/August), and end-season (November/December). The players were measured and tested within a week in each observation period. From the total sample, 53, 105, and 53 under-13 players completed observations at pre-, mid-, and end-season, respectively; 67, 102, and 43 under-15 players completed observations at pre-, mid-, and end-season, respectively; 31, 32, and 23 under-17 players completed observations at pre-, mid-, and end-season, respectively.

Hence a total of 509 measurements were considered. In addition, data from consecutive seasons were grouped by season to adjust for variation between seasons in the outcomes of interest.

Players were engaged in formal youth basketball training programs and competed in the state-level competition supervised by the local federation. All players trained at least three times a week (1.5–2.5 h/training day) and played a match most of the weekends over a 9-month competitive season. No players reported moderate or more severe injuries during 6-months before the testing. We grouped players into five age categories (under-13, under-15, and under-17) according to birth date and the date of assessment (for example, a player who would complete 13 years was classified as under 13, while a player who would complete 14 years in the same season was classified as under 15). The state basketball federations supervise youth basketball competitions in Brazil. In the present sample, players were engaged in official competitions in São Paulo and Santa Catarina, promoted by the *Federação Paulista de Basketball* and *Federação Catarinense de Basketball,* respectively. Clubs' programs run traditionally from February to July and August to November, completing nine months each season. Data were collected at each basketball club facility.

Players and their parents or legal guardians were informed of the nature of the study, the participation was voluntary, and they could withdraw from the study at any time. The study was approved by the Research Ethics Committee of the Federal University of Santa Catarina and by the Research Ethics Committee of the University of Campinas. Both athletes and their legal guardians provided written informed consent.

### 2.2 Procedures

#### 2.2.1 Anthropometric measurements

We considered anthropometry measurements taken by a single experienced observer following standardized procedures (26), including stature and body mass. Intra-observer technical measurement errors were 0.25 cm for stature and 0.42 kg for body mass (27).

#### 2.2.2 Chronological age and maturity status

Chronological age was considered to the nearest 0.1 years by subtracting a birth date from the testing date. The sex-specific maturity offset equations were used to estimate age at peak height velocity (PHV) based on the age and stature prediction model (28). The prediction model calculates the distance from PHV by subtracting the estimation from chronological age, i.e., the offset. With the offset estimation, we can derive each player's age at PHV. Often overlooked, the offset equations estimate timing (i.e., the age at which a given pubertal milestone is reached). However, the interest in interpreting young athletes' performance and development lies in tempo information, i.e., the rate of within-person progression through maturation stages (29). To interpret variation in maturity status between individuals, we compared the estimates of timing obtained with the sex-specific offset equations against the population references based on meta-analysis estimations (14). Hence, we compared the players' estimated age at PHV against a sex-specific reference age at PHV derived from a meta-analysis of longitudinal growth studies (16). Details of our procedure are available elsewhere (14). Then we classiﬁed the young basketball players as follows: *early maturers* (*n* = 90), when the estimated age at PHV was lower than the reference age at PHV by more than six months; *average maturers* (*n* = 52) when players' estimated age at PHV was within plus/minus six months of the reference age at PHV; *late maturers* (*n* = 5), when estimated age at PHV was higher than the reference age at PHV by more than six months.

Nevertheless, the limitations of the maturity offset protocol are assumed in our analysis (19), particularly at the extremes of the observed age range where bias is likely to be signiﬁcant (30). Therefore, we considered the maturity status of players from the under-13 for female players and under-13 and under-15 age groups for male players. Female under-15 and female and male under-17 players were categorized as *not classified* (*n* = 35). We assume our interpretations about the influence of maturity status on players across the ages that the offset protocol is less limited, i.e., the ages around the PHV (30).

#### 2.2.3 Onset of specialization in basketball

The age of specialization in basketball was considered as the self-reported age when athletes started formal year-round participation in a single “signature” sport (basketball), including training and competition in basketball, under the supervision of a coach within a youth basketball program registered in the state basketball federation, and with no participation in practice and competition in other organized sport (14). Hence, we follow a conceptual approach to specialization as year-round participation in a single “signature” sport, limited involvement in potential sport alternatives, and deliberate focus on training and development to pursue elite status (10, 13–15). The onset of specialization in basketball was defined by considering two biological maturation milestones, the age of initiation of the pubertal growth spurt and the age at PHV (14). Based on available longitudinal data from growth studies in the general population (16), the sex-specific reference age of the biological milestones was estimated using a meta-analysis fitted with multilevel models. The reference age of initiation of the pubertal growth spurt and age at PHV was 9.4 [95% Credible Interval (CI) 9.0 to 9.8] years and 11.1 (95% CI 10.8 to 11.5) years for females and males, respectively. The reference age at PHV was 11.9 (95% CI 11.8 to 12.0) years and 13.9 (95% CI 13.8 to14.0) years for females and males, respectively. Hence, the onset of specialization in basketball for young basketball players was classified as follows: pre-puberty specialization (i.e., early specialization), when players start their specialization in basketball before the reference age of onset of pubertal growth (*n* = 84); mid-puberty, when players began basketball specialization between the references for the onset of pubertal growth and the age of PHV, i.e., during pubertal growth (*n* = 60); late-puberty, when the start of specialization in basketball occurred after the reference age at pubertal growth (*n* = 13). For the cases where it was impossible to retain the onset of specialization, we classified players as unknown (*n* = 25).

The present study did not consider deliberate play (3) and informal participation in other sports before or after the specialization onset age in basketball. Therefore, similarly to our earlier observations with cross-sectional data (14), it was assumed that the limits of our data to describe the continuum of sport participation of the sample and caution is advised in interpreting the data.

#### 2.2.3 Physical fitness assessment

To describe players' basketball-specific physical fitness levels, we used the vertical jump with the countermovement jump (31), a short-term maximal running protocol with changes of direction, the line drill test (32), and intermittent endurance test, the yo-yo intermittent recovery level 1 test (yo-yo IR1) (33). Details about the present research project's physical fitness assessments and reliability estimates are available elsewhere (19, 27, 32). The height of the best countermovement jump was retained to the nearest centimeter. Each time performance in the line drill test was recorded in seconds. The covered distance in the yo-yo IR1 was measured in meters. Based on the sum of the z-scores of each physiological measurement, we estimated a score of overall physical fitness, i.e., physical fitness score (lower-limb explosive strength, agility and anaerobic power, and intermittent endurance) (20, 34). Given that lower times indicate better performance, the z-scores were reversed for the line drill test performance.

##### 2.2.3.1 Statistical models

The repeated observations of each player across a season and multiple seasons present an example of a complex hierarchical structure. A multilevel modeling approach in a fully Bayesian framework (35, 36) was used to cope with a complex data structure with an imbalanced sample size and heterogeneity among and between players. Readers unfamiliar with Bayesian methods may be surprised that we do not report significance tests in our results. In its place, we will use a direct probabilistic interpretation of the models' parameters to simulate predictions and assess the quality of model fit to data (37).

We used two model structures to examine changes within a competitive season and developmental changes during adolescence.

#### 2.2.4 Modeling changes within a competitive season

Varying intercept and varying slope models were fitted to the repeated measures data, allowing for the possibility of varying intercepts (i.e., pre-season values) and slope (changes in players' outcomes across mid- and end-season) by players. In addition, sex was included as a population-level (also referred to as ﬁxed effect) due to the difﬁculty of estimating the between-group variation when the number of groups is small (38). Furthermore, within- and between-group variation was incorporated in the model on the players' physical fitness changes across the competitive season. To capture variation in physiological responses by sex, we included an interaction term between sex and changes in players' outcomes across mid- and end-season. Also, to capture differences in physical fitness by sex, we allow players by sex to vary by the onset of specialization and age group maturity status. The group-level effect terms (also called random effects) and data-level terms (also called level-1 residuals) were drawn from normal distributions with variances to be estimated from the data. Note that some of these variables include “Unknown” or “Not classified” values and keep these values as separate levels in the model. We partially pool within each group to allow the model to pick up trends in cases with insufficient data or missing information to project the estimates onto the imbalanced repeated measures data.

When modeling the yo-yo IR1 and the overall physical fitness score responses, we included body mass (standardized) as a population-level effect to partition the influence of size on physical fitness outcomes, particularly long-term intermittent maximal performance (39). However, there was no need to include body dimensions for the short-term maximal outputs, as the influence of size on performance was neglectable.

#### 2.2.5 Modeling developmental changes

We fitted a basic three-level polynomial growth model curve (40) to model physical fitness indicators against chronological age. The model describes each player's successive measurements over time, deﬁning the player's change at each measurement point and its variation (level-1), differences in trajectories between players, and its variation (level-2), and differences in trajectories between players grouped by specialization onset, and its variation (level-3). To describe potential non-linear developmental changes during adolescence, we considered time (i.e., chronological age) coefficients up to the quadratic terms. In addition, we allowed for developmental trajectories to vary between players (level-2) and between players grouped by specialization onset.

#### 2.2.6 Prior distributions

For interpretative convenience and to speed up computation, we standardized the outcomes by subtracting the mean and dividing by two standard deviations (38). Given that young players' physical fitness outcomes tends to be heterogeneous and the available imbalanced repeated measures data, we were intentionally conservative in our interpretations. Hence, we used weakly informative priors to regularize our estimates. We used multivariate normal priors (0,5) for the population-level parameter (i.e., intercept and slopes) and exponential (1) priors for the group-level parameters. For the data-level residuals, we used the default prior, Student-t (3, 0, 2.5) (41).

#### 2.2.7 Statistical software, code repository, and reproducibility

The length of the chains and warm-up was sufficient to achieve convergence and obtain a reasonable, effective sample size. We ran four chains for 2,000 iterations with a warm-up length of 1,000 iterations for each model. The models were inspected and validated using posterior predictive checks (42). The Bayesian multilevel models were fitted using R statistical language (43) with the “brms” package (41), which call Stan (44).To extract the posterior samples and visualize the results, we used the “tidybayes” (45) and “ggplot2” (46) packages. The data, codes, and details about models specifications and posterior predictive checks are available as supplementary material (https://osf.io/2gfw5/).

## 3 Results

Characteristics of the sample at pre-season, as reference for description, are summarized in Table 1. Under-13 and under-15 players were mostly classified as early or average maturers with an approximately 2 to 1 distribution of cases, respectively. The distribution of players by the onset of specialization in youth basketball within age groups is summarized in Table 2.

**Table 1:** Descriptive statistics of the sample at pre-season by age group and sex.

	Under-13		Under-15		Under-17	
	Female	Male	Female	Male	Female	Male
Chronological age, years	12.5 (0.5)	12.6 (0.3)	14.2 (0.6)	14.2 (0.6)	16.0 (0.6)	15.9 (0.5)
Maturity offset, years	0.92 (0.67)	−0.44 (0.57)	^b^	1.04 (0.83)	^b^	^b^
Estimated age at PHV^a^, years	11.6 (0.5)	13.1 (0.5)	^b^	13.1 (0.5)	^b^	^b^
Stature, cm	163.3 (9.5)	165.4 (11.0)	164.3 (5.6)	176.6 (11.0)	167.7 (3.9)	186.5 (7.5)
Body mass, kg	56.9 (13.6)	56.8 (16.1)	58.0 (8.5)	65.3 (13.3)	59.2 (8.4)	80.3 (11.0)
Countermovement jump, cm	24.3 (3.9)	31.7 (6.7)	25.0 (3.9)	35.6 (5.8)	25.7 (4.1)	38.9 (6.0)
Line Drill test, *s*	37.36 (1.88)	34.71 (3.37)	35.67 (1.93)	33.57 (3.70)	36.29 (1.82)	31.70 (3.09)
Yo-yo recovery test – level I, m	372.7 (82.2)	516.4 (305.6)	516.2 (175.7)	899.2 (382.8)	528.0 (171.6)	1203.3 (348.0)
Performance score, *z*-score sum	−1.41 (0.59)	−0.25 (1.47)	−0.92 (0.63)	0.65 (1.11)	−0.91 (0.69)	1.74 (0.96)

^a^ PHV, peak height velocity.

^b^ Female under 15 and both female and male under 17 players were not classified by maturity status due to the lack of validity of the offset estimations.

**Table 2:** Distribution Of players by the onset of specialization within an age group and sex in the sample of young Brazilian players.

	Under-13		Under-15		Under-17		Total
	Female	Male	Female	Male	Female	Male	
Pre-puberty specialization	5	34	3	34	2	5	84
Mid-puberty specialization	12	21	9	15	1	3	60
Late-puberty specialization	5	Not possible	3	2	1	2	13
Unknown in the sample	0	7	0	12	0	6	25

**Figure 1:**
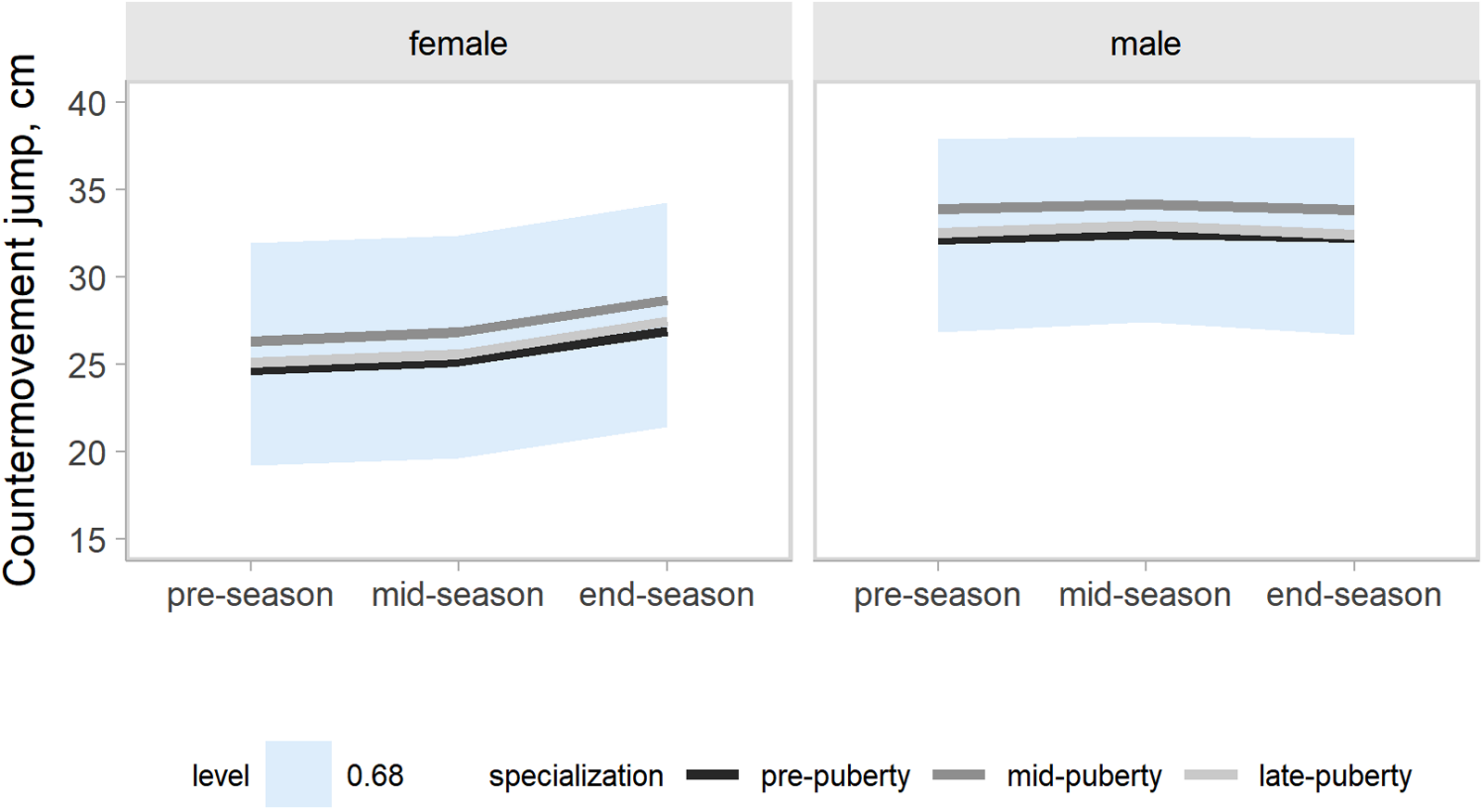
Changes in countermovement jump performance for young females and male basketball players within a basketball season by the onset of specialization. The shaded area represents the 68% credible interval, similar to a standard deviation.

**Figure 2:**
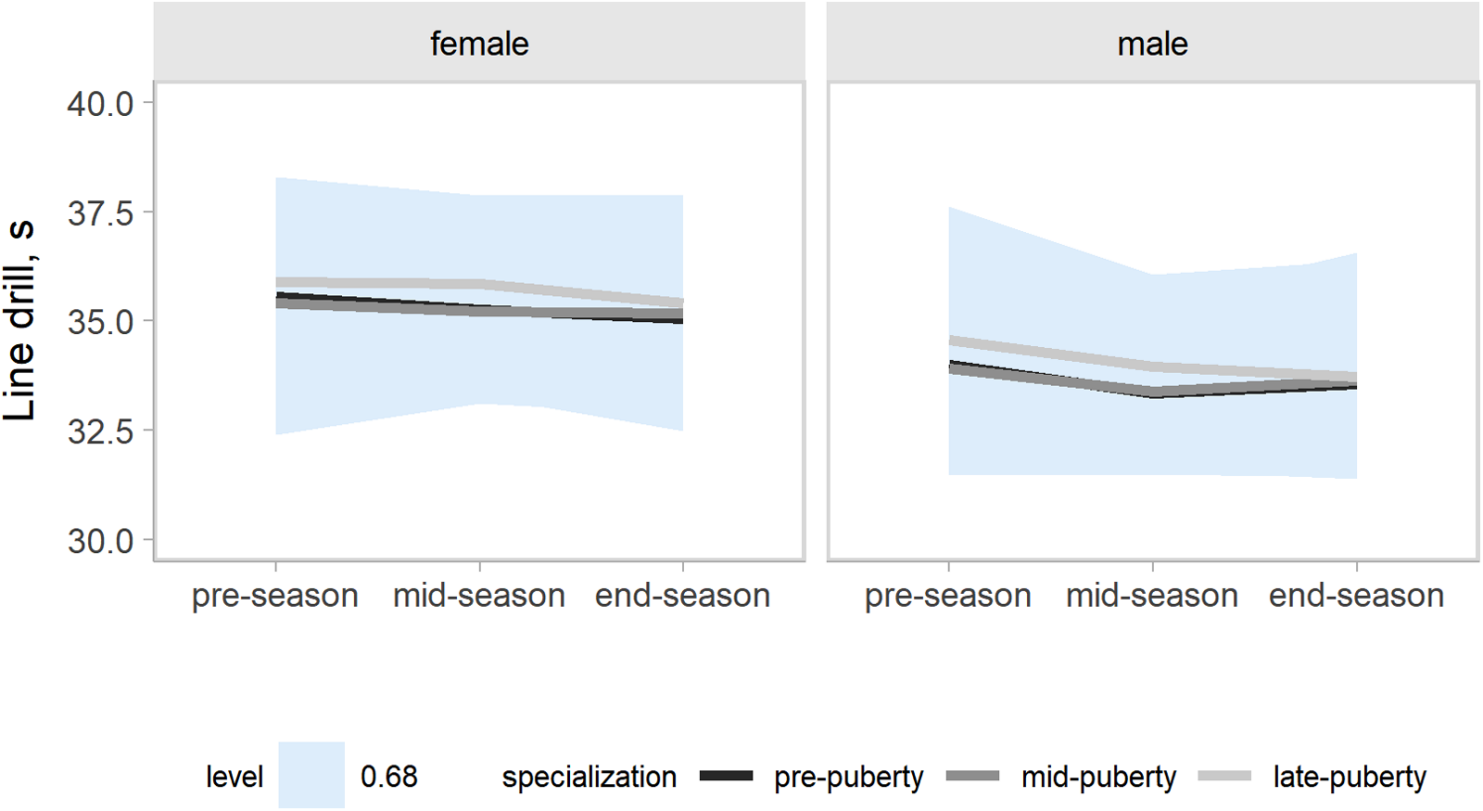
Changes in line-drill performance for young females and male basketball players within a basketball season by the onset of specialization. The shaded area represents the 68% credible interval, similar to a standard deviation.

**Figure 3:**
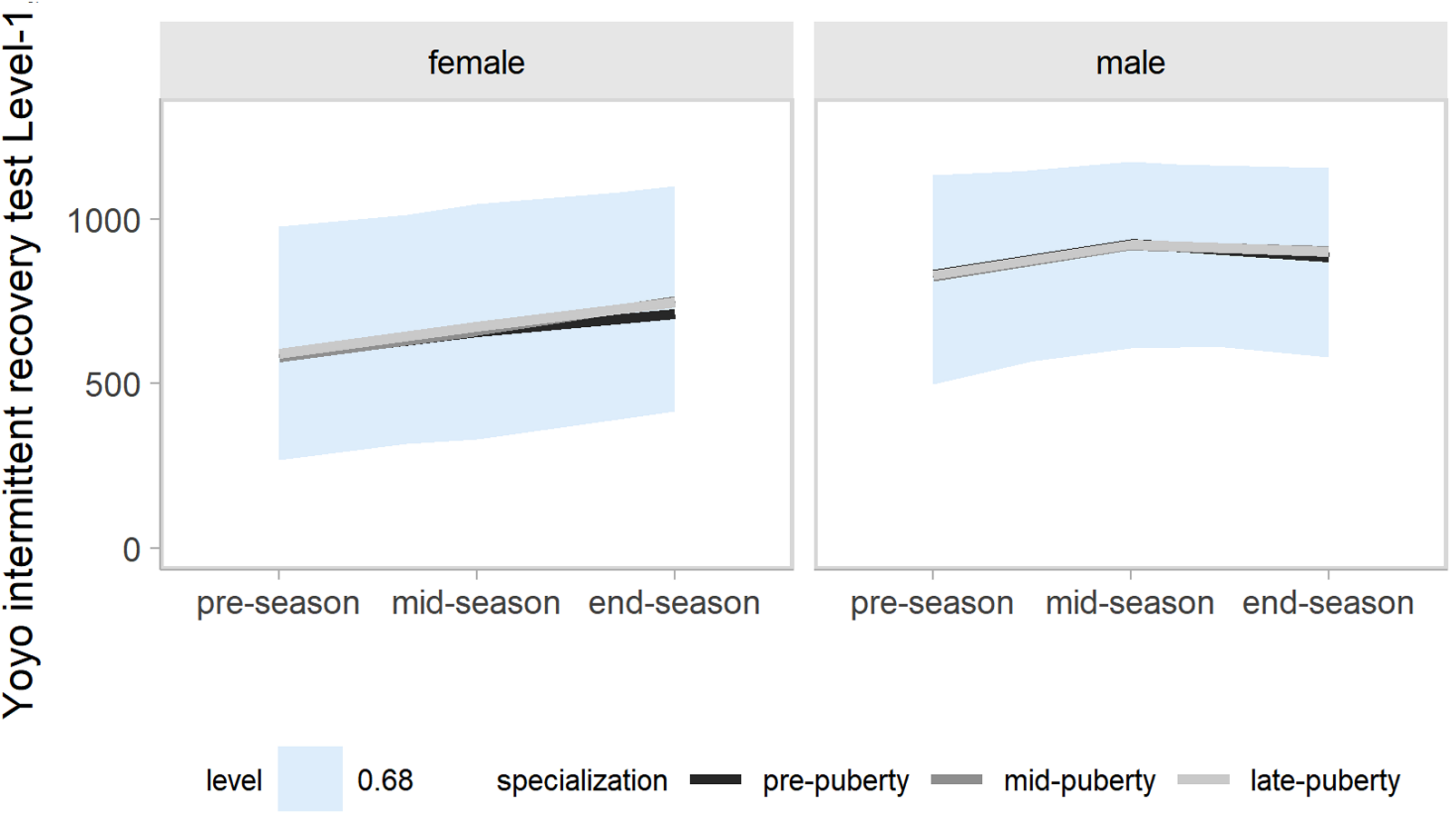
Changes in Yo-yo intermittent recovery test level 1 performance for young females and male basketball players within a basketball season by the onset of specialization. The shaded area represents the 68% credible interval, similar to a standard deviation.

**Figure 4:**
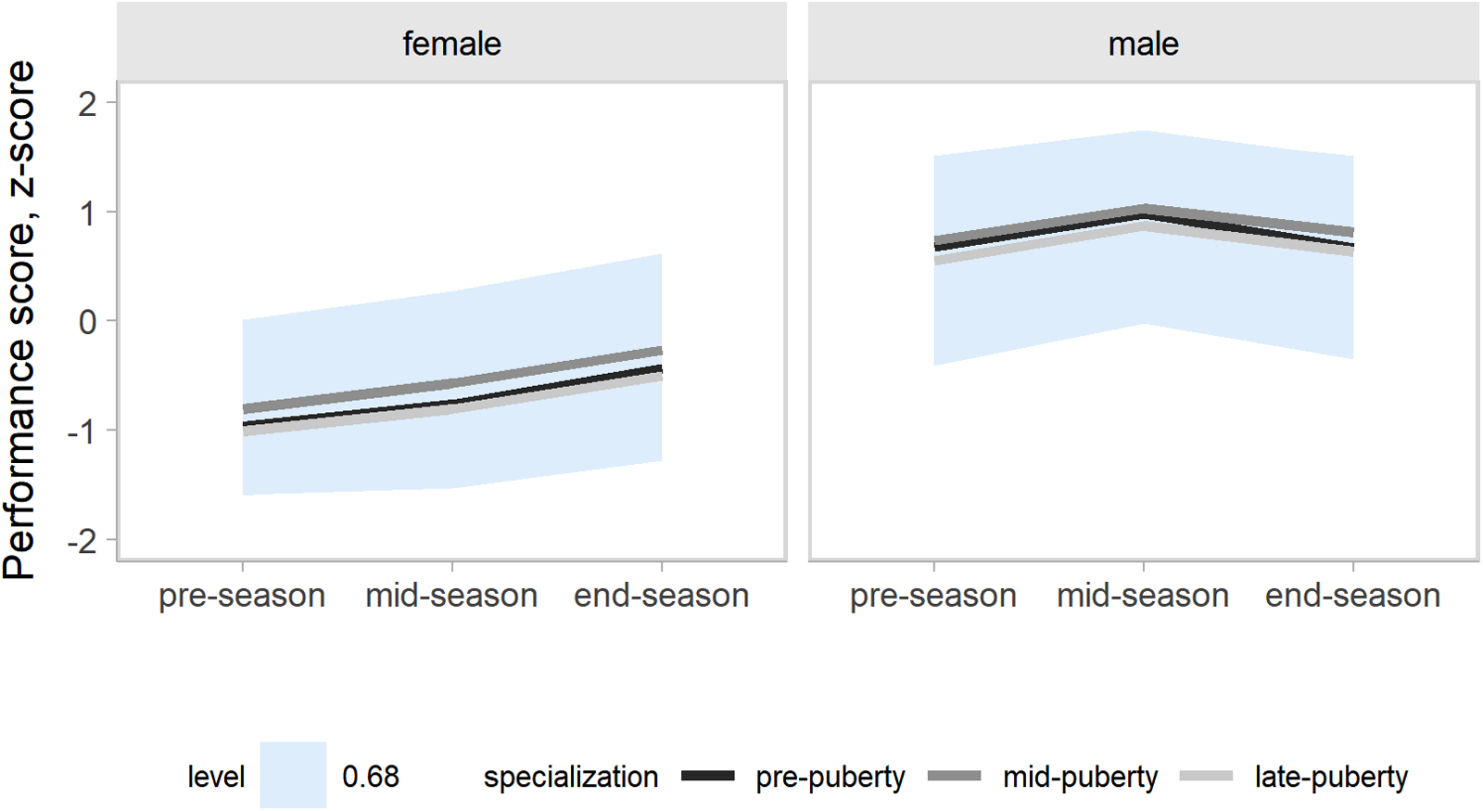
Changes in overall basketball-specific physical fitness index for young females and male basketball players within a basketball season by the onset of specialization. The shaded area represents the 68% credible interval, similar to a standard deviation.

Our models accounted for variation in the outcomes changes across a competitive season associated with age group, maturity status, and the onset of specialization. Hence, the effects of target groups can be interpreted as accounting for the other group effects. In the present study, our main focus was the contrasts by the onset of specialization. Predictions and uncertainty (68% credible intervals, i.e., approximately a standard deviation) of countermovement jump (Figure 1), Line drill test (Figure 2), yo-yo IR1 (Figure 3), and overall fitness score (Figure 4) changes across a competitive season are plotted. In addition, we contrasted playerś physical fitness predictions by the onset of specialization within sex. We observed no substantial variation by the onset of specialization for female and male players in the basketball-specific physical fitness changes across a competitive season. However, the trend of changes across a competitive season varied by sex. Female players showed slight improvements in countermovement jump, yo-yo IR1, and overall performance score. In contrast, male players maintained their physical fitness levels throughout the competitive season. Overall, older players presented higher values for the indicators of physical fitness across the competitive season. There was no substantial variation in the physical fitness outcomes by maturity status in the responses across the competitive season. Supplementary plots of predictions of changes in the physical fitness outcomes across a competitive season by age group and maturity status within sex are available at https://osf.io/2gfw5/.

**Figure 5:**
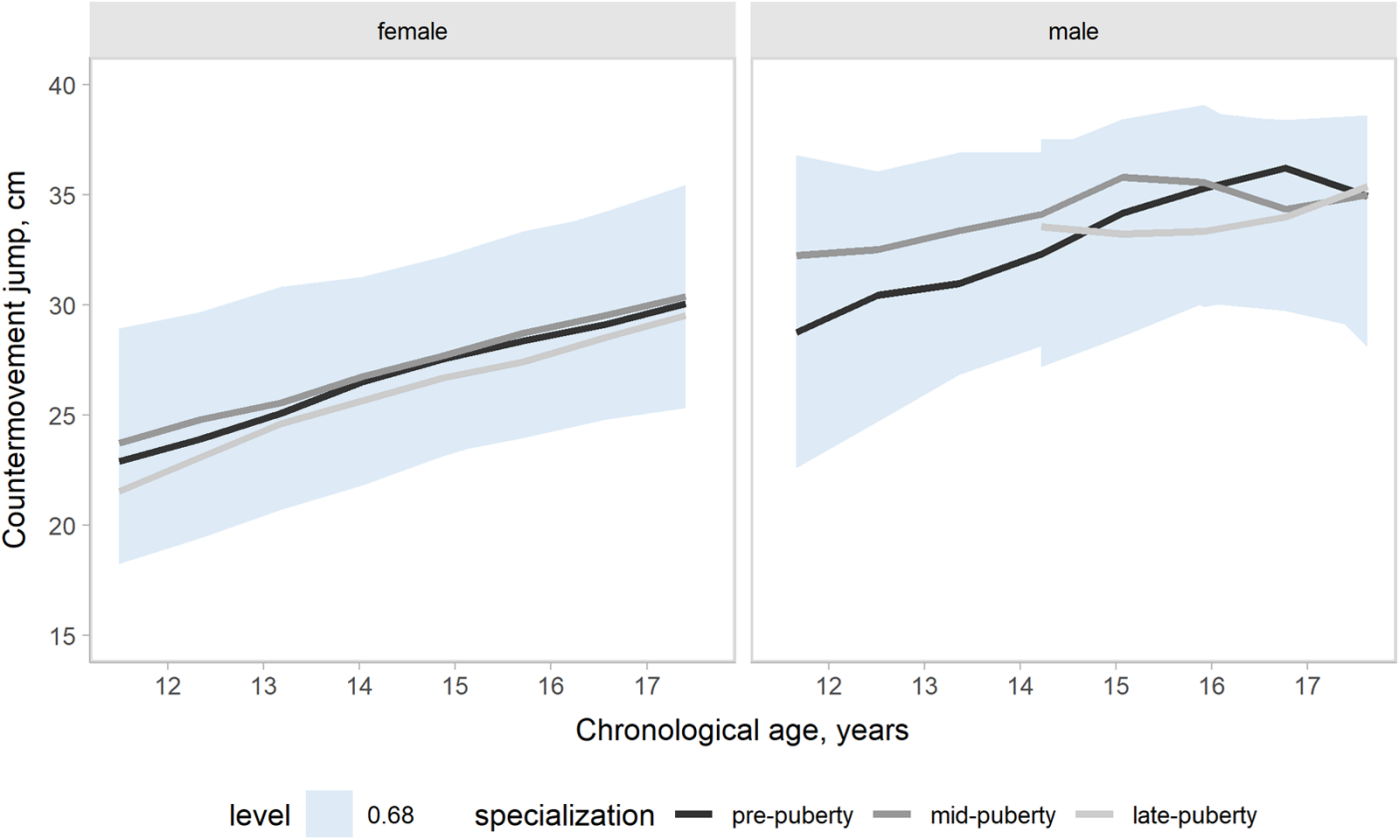
Developmental changes in countermovement jump performance for young females and male basketball players by specialization onset. The shaded area represents the 68% credible interval, similar to a standard deviation.

**Figure 6:**
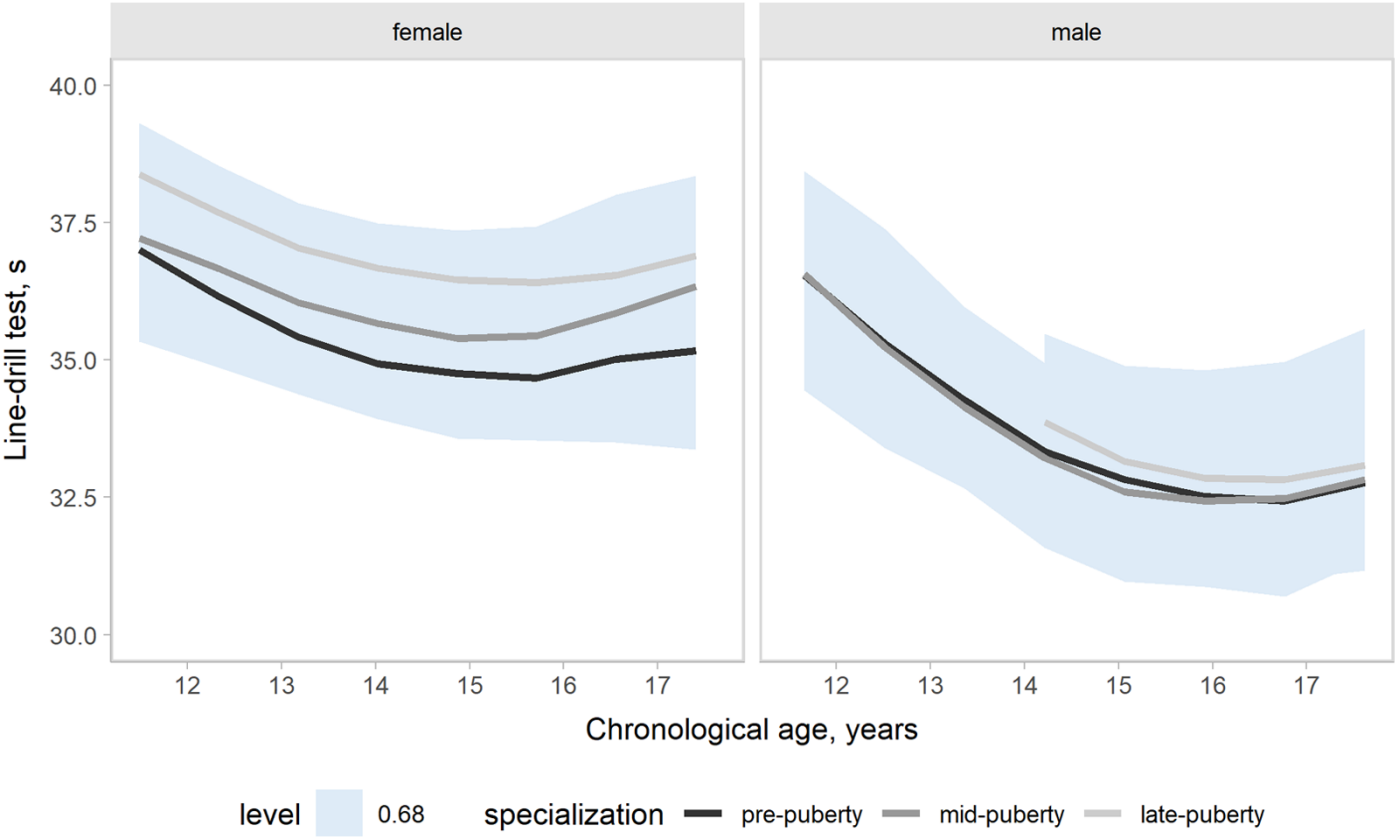
Developmental changes in line-drill performance for young females and male basketball players by specialization onset. The shaded area represents the 68% credible interval, similar to a standard deviation.

**Figure 7:**
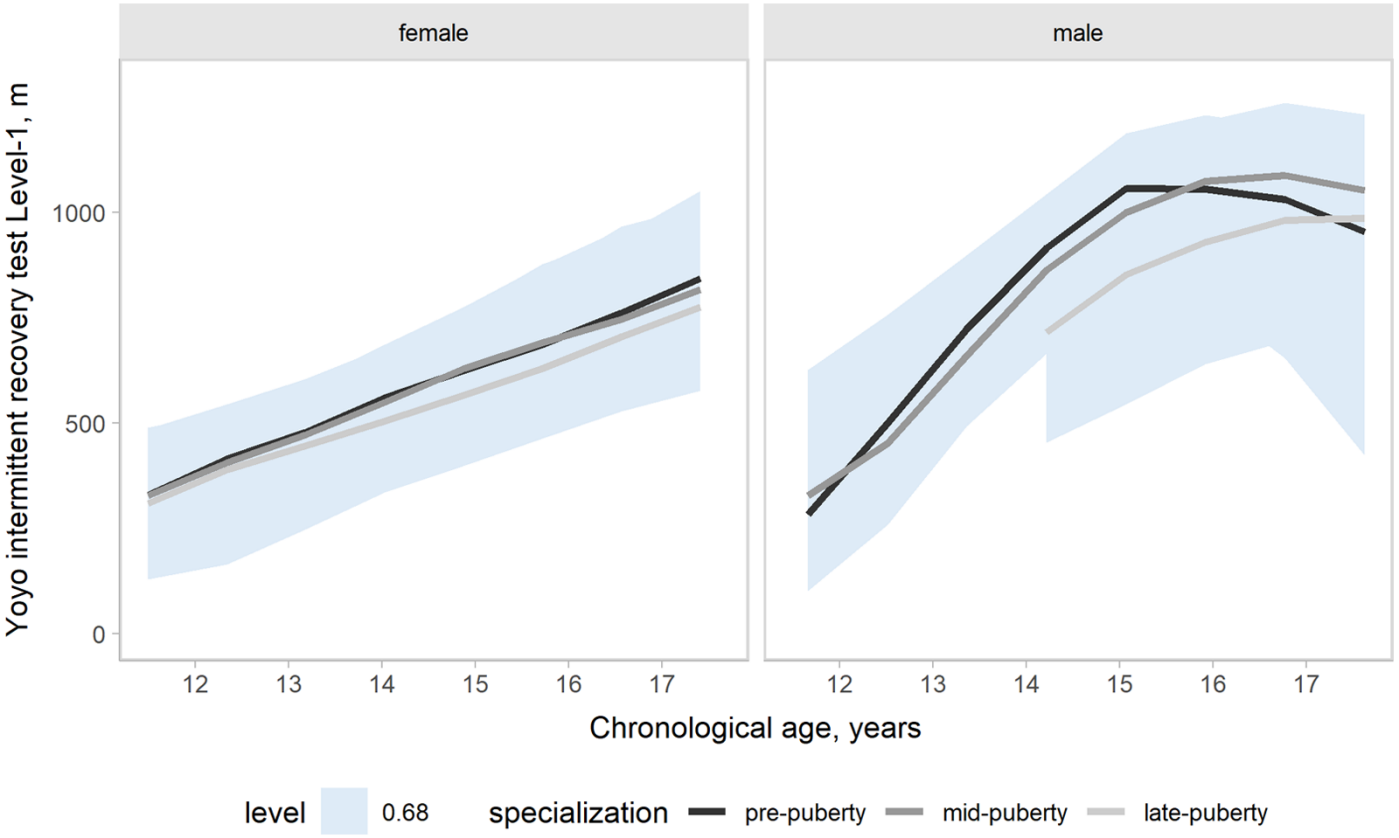
Developmental changes in Yo-yo intermittent recovery test level 1 performance for young females and male basketball players by specialization onset. The shaded area represents the 68% credible interval, similar to a standard deviation.

**Figure 8:**
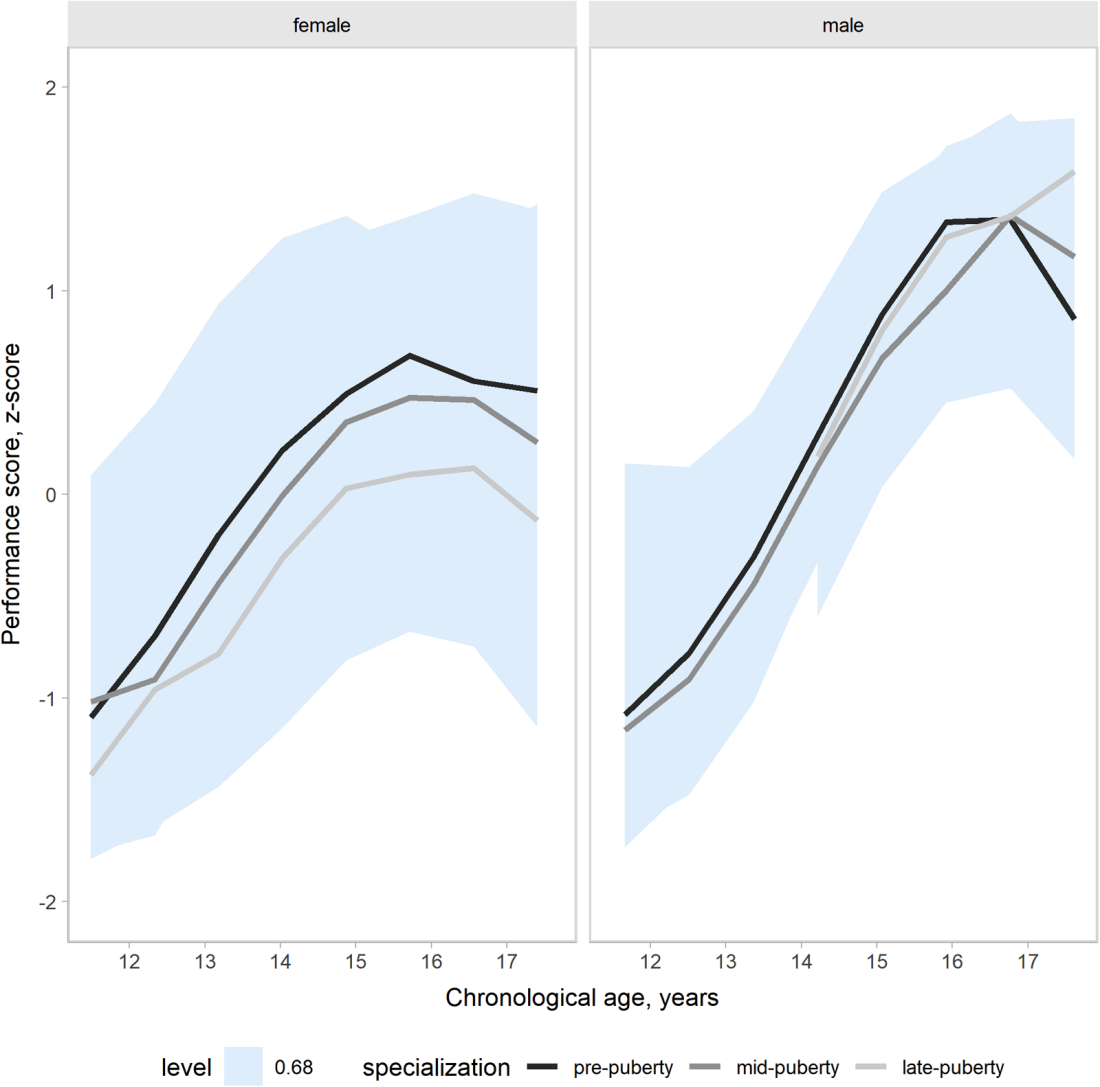
Developmental changes in overall basketball-specific physical fitness performance index for young females and male basketball players by specialization onset. The shaded area represents the 68% credible interval, similar to a standard deviation.

Our three-level growth models accounted for variation in the outcomes changes between 11 and 17 years, accounting for the potential influence of the specialization onset. Predictions and uncertainty (68% credible intervals) of countermovement jump (Figure 5), Line drill test (Figure 6), yo-yo IR1 (Figure 7), and overall fitness score (Figure 8) developmental changes are plotted, contrasting the onset of specialization within sex. Notably, there was no substantial variation by the onset of specialization for both female and male players in the basketball-specific physical fitness developmental changes. However, we observed differences in the magnitude and pattern of developmental changes in physical fitness between female and male players when aligned by chronological age. The plots contrasting developmental changes by sex for countermovement jump (Supplementary Figure S9), Line drill test (Supplementary Figure S10), yo-yo IR1 (Supplementary Figure S11), and overall fitness score (Supplementary Figure 12) are available at https://osf.io/2gfw5/.

## 4 Discussion

In the present study, we tested the assumption supporting early specialization, stating that there are basketball-specific physical fitness advantages of early specialization in young players (1, 6). The most interesting observation in this study is that players who specialize early in basketball (i.e., pre-puberty specialization) do not appear to have an advantage in basketball-specific physical fitness levels development. Conditional on our data, early specialization in youth basketball does not provide an advantage in developing physical across a season. Improvements in basketball-specific performance across a competitive season were apparent only for female players. In contrast, male players appear to maintain their physical fitness levels across a competitive season, adjusting for age group and estimated maturity status. Physical fitness developmental advantages were also not observed for players with early specialization. Therefore, young basketball players who specialize before pubertal growth (early) do not appear to have better physical fitness and develop faster than those who specialize during or after the pubertal growth period.

The growth characteristics of the present sample of Brazilian female and male adolescent basketball players were consistent with other reports with heterogeneous samples of young athletes (47) and young basketball players (19, 22, 23, 48, 49). Overall, the somatic indicator suggests that the sample of female and male players across the age span of pubertal growth had an advanced maturity status. Nevertheless, caution is warranted when interpreting and generalizing the maturity status of young athletes based on somatic maturity indicators, as the prediction equations have limited validity (19, 30).

Sport specialization, in particular, early specialization, is a key issue in organized youth sports. Despite the interest and concerns surrounding children's early exposure to intense sports competition, little scientific information supports or refutes these risks (7, 50–52). It has been argued that there is a lack of evidence that specialization before puberty is necessary to achieve elite status and that specialization before puberty is more likely to be detrimental (53). Nevertheless, these interpretations are mainly based on inconsistent evidence with a potential sample bias (54). On the other hand, early specialization appears to have become the common *modus operandi* in competitive youth sports (1, 7, 13). The assumption that early sport specialization provides a physiological advantage for future athletic success (6) may result from interpreting the deliberate practice theory (2) applied to youth sports. Conditional to our data and models, early specialization in youth basketball (i.e., pre-puberty specialization) does not provide an advantage in developing basketball-specific physical fitness or improved responses within a season.

Another issue that biases the discussion about sports specialization is the inconsistent definition of early specialization (9–11). In some cases, the operational definition of early specialization is based on the stages of developmental models, establishing the age of 12 as a reference (55). However, in other reports, it is unclear how early (or late) specialization is operationalized (11). Therefore, to understand the mechanisms behind early (and late) specialization and why it is potentially harmful or beneﬁcial, first, it must be established what early specialization is and the best methods to assess it (9).

Youth sports participation and specialization can be conceptualized as a continuum. We propose that specialization can be defined and interpreted relative to biological maturation milestones describing the pubertal growth period, i.e., the age of initiation of the pubertal growth spurt and the age at peak height velocity (PHV). We used a meta-analysis to establish the references conditional on general population growth patterns (14). We defined, in general, specialization as year-round participation in a single “signature” sport, with limited involvement in potential sport alternatives, with a deliberate focus on training and development in the pursuit of elite status (10, 13, 14).

We believe that the main characteristics of young players' development were captured, allowing for variation between sports specificities and contexts. Hence, players who attain the conditions defined as specialization before about nine years and 11 years for girls and boys, respectively, are considered as experiencing early specialization. On the other hand, late specialization may be interpreted as those players who attain the conditions defined as specialization after the age at PHV, about 12 years for girls and 14 years for boys.

The study of the development of physical fitness levels of basketball players during a basketball-competitive season is limited, and even more in young players (24, 25). The interpretation of the data is limited by the small number of studies, each with small sample sizes and measurement errors associated with the performed tests (56–59). As a result, the changes (decreases or improvements across a season by competitive level or starters vs. nonstarters) are trivial or inconclusive. Nevertheless, the observations with young basketball players from the Australian national- and state-level developmental programs showed a trend of improvement in physical fitness assessments across a season (25), particularly in the Line drill test (24). Also, the physical fitness changes within a season vary by sex and competitive level among young Australian basketball players (24, 25). The young male players in the present sample maintained their physical fitness levels across a competitive season. In contrast, the female players showed a slight improvement in their fitness within a season. Nevertheless, given the variability in our predictions, a conservative interpretation and generalization should be taken.

Longitudinal data considering physical fitness development in youth basketball is scarce (20, 60, 61). Conditional on the data, physical fitness outcomes improved, on average, with age 11 to 17 for both female and male players. On average, young male players showed higher values in fitness indicators than young female players. It was apparent that players showed increased rates of fitness development overlapping with the period of pubertal growth. For female players, a leveling-off in fitness development was apparent earlier than for male players, particularly visible in the Line drill test. Sex-related differences in the timing and magnitude of the development of physical fitness outcomes are likely associated with sexual dimorphism during pubertal growth that underlies physiological functions (4, 62). Sex-related differences in fitness became apparent as girls, on average, attain biological milestones in puberty earlier than boys, albeit the large between-individual variation in the timing and tempo of biological maturation (4).

Our study provided valuable data regarding the interpretation of seasonal variations and developmental changes in basketball-specific physical fitness variables in adolescent basketball players. Nevertheless, the available unbalanced sample size, context specificities, and maturity indicator limitations warrant caution when compared with other adolescent basketball players. However, the multilevel modeling in a fully Bayesian framework allows dealing with non-representative and imbalanced samples, with hierarchical sources of variation and cross-classiﬁed nesting (38). Bayesian methods comprise samples from the joint posterior density of the parameters (37). It allows for direct probabilistic interpretation of credible (also referred to as compatibility or uncertainty intervals) intervals and posterior probabilities (63). Bayesian methods should be of interest to those concerned with estimations of very small effects, typical of within-athletes changes in response to training, measured with noisy measurements, as is often the case with physical fitness outcomes.

Based on our data and models, early specialization before the onset of pubertal growth does not provide an advantage in basketball-specific physical fitness development across a season in youth basketball. Hence, the argument/myth that early sport specialization provides a fitness advantage for future athletic success does not hold. Overall, young athletes, coaches, and interested stakeholders should be conservative in their expectations of physical fitness improvements across a season among young basketball players. Furthermore, we provide an operational framework to interpret specialization related to biological maturation milestones.

## Data Availability

The datasets presented in this study can be found in online repositories. The names of the repository/repositories and accession number(s) can be found below: https://osf.io/2gfw5/.

## References

[1] Brylinsky J. Practice makes perfect and other curricular myths in the sport specialization debate. J Phys Educ Recreation Dance. (2010) 81(8):22–5. 10.1080/07303084.2010.10598522

[2] Ericsson KA Krampe RT Tesch-Römer C. The role of deliberate practice in the acquisition of expert performance. Psychol Rev. (1993) 100(3):363–406. 10.1037/0033-295X.100.3.363

[3] Côté J Murphy-Mills J Abernethy B. The development of skill in sport. In: Hodges NJ Williams AM, editors. Skill acquisition in sport: Research, theory and practice. 2nd edn ed. Abingdon, Oxon: Routledge; 2012. p. 295–312.

[4] Armstrong N. Development of the youth athlete. Abingdon, Oxon: Routledge (2019). 250.

[5] Baker J Young B. 20 Years later: deliberate practice and the development of expertise in sport. Int Rev Sport Exerc Psychol. (2014) 7(1):135–57. 10.1080/1750984X.2014.896024

[6] Kaleth AS Mikesky AE. Impact of early sport specialization. J Phys Educ Recreation Dance(2010) 81(8):29–37. 10.1080/07303084.2010.10598524

[7] Herman DC Nelson VR Montalvo AM Myer GD Brenner JS DiFiori JP Systematic review of health organization guidelines following the AMSSM 2019 youth early sport specialization summit. Sports Health. (2022) 14(1):127–34. 10.1177/19417381211056089 34668459 PMC8669928

[8] Waldron S DeFreese JD Register-Mihalik J Pietrosimone B Barczak N. The costs and benefits of early sport specialization: a critical review of literature. Quest. (2020) 72(1):1–18. 10.1080/00336297.2019.1580205

[9] Mosher A Fraser-Thomas J Baker J. What defines early specialization: a systematic review of literature. Front Sports Active Living. (2020) 2(164):596229. 10.3389/fspor.2020.596229 PMC7739675 33345176

[10] DiSanti JS Erickson K. Youth sport specialization: a multidisciplinary scoping systematic review. J Sports Sci. (2019) 37(18):2094–105. 10.1080/02640414.2019.1621476 31135271

[11] Mosher A Till K Fraser-Thomas J Baker J. Revisiting early sport specialization: what's The problem? Sports Health. (2022) 14(1):13–9. 10.1177/19417381211049773 34651518 PMC8655480

[12] Pasulka J Jayanthi N McCann A Dugas LR LaBella C. Specialization patterns across various youth sports and relationship to injury risk. Phys Sportsmed. (2017) 45(3):344–52. 10.1080/00913847.2017.1313077 28351225

[13] Baker J Cobley S Fraser-Thomas J. What do we know about early sport specialization? Not Much! High Abil Stud. (2009) 20(1):77–89. 10.1080/13598130902860507

[14] Lima AB Nascimento JV Leonardi TJ Soares AL Paes RR Gonçalves CE Deliberate practice, functional performance and psychological characteristics in young basketball players: a Bayesian multilevel analysis. Int J Environ Res Public Health. (2020) 17(11):4078. 10.3390/ijerph17114078 32521647 PMC7312187

[15] Coakley J. The “logic” of specialization. J Phys Educ Recreation Dance. (2010) 81(8):16–25. 10.1080/07303084.2010.10598520

[16] Malina RM Bouchard C Beunen G. Human growth: selected aspects of current research on well-nourished children. Annu Rev Anthropol. (1988) 17(1):187–219. 10.1146/annurev.an.17.100188.001155

[17] World Association of Basketball Coaches. Coaches manual: Mini-basketball: FIBA-WABC; 2016. Available at: https://www.fiba.basketball/documents/Mini-Basketball-English.pdf.

[18] Drinkwater EJ Pyne DB McKenna MJ. Design and interpretation of anthropometric and fitness testing of basketball players. Sports Med. (2008) 38(7):565–78. 10.2165/00007256-200838070-00004 18557659

[19] Carvalho HM Gonçalves CE Collins D Paes RR. Growth, functional capacities and motivation for achievement and competitiveness in youth basketball: an interdisciplinary approach. J Sports Sci. (2018) 36(7):742–8. 10.1080/02640414.2017.1340654 28604286

[20] Carvalho HM Leonardi TJ Soares ALA Paes RR Foster C Gonçalves CE. Longitudinal changes of functional capacities among adolescent female basketball players. Front Physiol. (2019) 10(339):339. 10.3389/fphys.2019.00339 31019466 PMC6459046

[21] Arede J Esteves P Ferreira AP Sampaio J Leite N. Jump higher, run faster: effects of diversified sport participation on talent identification and selection in youth basketball. J Sports Sci. (2019) 37(19):2220–7. 10.1080/02640414.2019.1626114 31164046

[22] Ramos S Volossovitch A Ferreira AP Fragoso I Massuca LM. Training experience and maturational, morphological, and fitness attributes as individual performance predictors in Male and female under-14 Portuguese elite basketball players. J Strength Cond Res. (2021) 35(7):2025–32. 10.1519/JSC.0000000000003042 30741870

[23] Torres-Unda J Zarrazquin I Gravina L Zubero J Seco J Gil SM Basketball performance is related to maturity and relative age in elite adolescent players. J Strength Cond Res. (2016) 30(5):1325–32. 10.1519/JSC.0000000000001224 26439783

[24] Montgomery PG Pyne DB Hopkins WG Minahan CL. Seasonal progression and variability of repeat-effort line-drill performance in elite junior basketball players. J Sports Sci. (2008) 26(5):543–50. 10.1080/02640410701654298 18274951

[25] Drinkwater EJ Hopkins WG McKenna MJ Hunt PH Pyne DB. Characterizing changes in fitness of basketball players within and between seasons. Int J Perform Analy Sport. (2005) 5(3):107–25. 10.1080/24748668.2005.11868342

[26] Lohman TG Roche AF Martorell R. Anthropometric standardization reference manual. Abridged ed., Champaign, IL: Human Kinetics Books (1991).

[27] Soares ALA Kós LD Paes RR Nascimento JV Collins D Gonçalves CE Determinants of drop-out in youth basketball: an interdisciplinary approach. Res Sports Med. (2020) 28(1):84–98. 10.1080/15438627.2019.1586708 30835570

[28] Moore SA McKay HA Macdonald H Nettlefold L Baxter-Jones AD Cameron N Enhancing a somatic maturity prediction model. Med Sci Sports Exerc. (2015) 47(8):1755–64. 10.1249/MSS.0000000000000588 25423445

[29] Gonçalves CE Carvalho HM. Revisiting the relative age effect from a multidisciplinary perspective in youth basketball: a Bayesian analysis. Front Sports Active Living. (2021) 2(230):581845. 10.3389/fspor.2020.581845 PMC7884768 33604567

[30] Koziel SM Malina RM. Modified maturity offset prediction equations: validation in independent longitudinal samples of boys and girls. Sports Med. (2018) 48(1):221–36. 10.1007/s40279-017-0750-y 28608181 PMC5752743

[31] Bosco C Luhtanen P Komi PV. A simple method for measurement of mechanical power in jumping. Eur J Appl Physiol Occup Physiol. (1983) 50(2):273–82. 10.1007/BF00422166 6681758

[32] Carvalho HM Gonçalves CE Grosgeorge B Paes RR. Validity and usefulness of the line drill test for adolescent basketball players: a Bayesian multilevel analysis. Res Sports Med. (2017) 25(3):333–44. 10.1080/15438627.2017.1314296 28391721

[33] Bangsbo J. Fitness training in footbal - a scientific approach. Bangsvaerd: HO Storm (1994).

[34] Malina RM Ribeiro B Aroso J Cumming SP. Characteristics of youth soccer players aged 13–15 years classified by skill level. Br J Sports Med. (2007) 41(5):290. 10.1136/bjsm.2006.031294 17224444 PMC2659047

[35] Gelman A Carlin JB Stern HS Dunson DB Vehtari A Rubin DB. Bayesian Data analysis. Boca Raton, FL: Chapman & Hall/CRC Press (2013).

[36] McElreath R. Statistical rethinking: a Bayesian course with examples in R and stan. 2nd edition. Boca Raton, FL: Chapman and Hall/CRC Press (2020).

[37] McElreath R Koster J. Using multilevel models to estimate variation in foraging returns. Effects of failure rate, harvest size, age, and individual heterogeneity. Human Nat. (2014) 25(1):100–20. 10.1007/s12110-014-9193-4 24522975

[38] Gelman A Hill J. Data analysis using regression and multilevel/hierarchical models. Cambridge: Cambridge University Press (2007).

[39] Welsman J Armstrong N. Interpreting aerobic fitness in youth: the fallacy of ratio scaling. Pediatr Exerc Sci. (2019) 31(2):184–90. 10.1123/pes.2018-0141 30332906

[40] Goldstein H. Efficient statistical modelling of longitudinal data. Ann Hum Biol. (1986) 13(2):129–41. 10.1080/03014468600008271 3707042

[41] Bürkner P-C. Brms: an R package for Bayesian multilevel models using stan. J Stat Softw. (2017) 80:1–28. 10.18637/jss.v080.i01

[42] Gabry J Simpson D Vehtari A Betancourt M Gelman A. Visualization in Bayesian workflow. J R Stat Soc. (2019) 182(2):389–402. 10.1111/rssa.12378

[43] R Core Team. R: a language and environment for statistical computing. Vienna, Austria: R Foundation for Statistical Computing (2018). Available at: http://www.R-project.org/.

[44] Stan Development Team. Stan: A C++ Library for Probability and Sampling 2015. Available at: http://mc-stan.org/.

[45] Kay M. tidybayes: Tidy Data and Geoms for Bayesian Models 2021, Available at: http://mjskay.github.io/tidybayes/.

[46] Wickham H. Ggplot2: elegant graphics for data analysis. New York: Springer-Verlag (2016).

[47] Malina RM. Physical growth and biological maturation of young athletes. Exerc Sport Sci Rev. (1994) 22:389–433. 10.1249/00003677-199401000-00012 7925550

[48] Leonardi TJ Paes RR Breder L Foster C Gonçalves CE Carvalho HM. Biological maturation, training experience, body size and functional capacity of adolescent female basketball players: a Bayesian analysis. Int J Sports Sci Coach. (2018) 13(5):713–22. 10.1177/1747954118772489

[49] Carvalho HM Silva M Figueiredo AJ Gonçalves CE Philippaerts RM Castagna C Predictors of maximal short-term power outputs in basketball players 14-16 years. Eur J Appl Physiol. (2011) 111(5):789–96. 10.1007/s00421-010-1703-4 20981436

[50] Committee on Sports Medicine and Fitness. Intensive training and sports specialization in young athletes. Pediatrics. (2000) 106(1):154–7. 10.1542/peds.106.1.154 10878168

[51] Brenner JS LaBella CR Brookes MA Diamond A Sports specialization and intensive training in young athletes. Pediatrics. (2016) 138(3):e20162148. 10.1542/peds.2016-2148 27573090

[52] Fabricant PD Lakomkin N Sugimoto D Tepolt FA Stracciolini A Kocher MS. Youth sports specialization and musculoskeletal injury: a systematic review of the literature. Phys Sportsmed. (2016) 44(3):257–62. 10.1080/00913847.2016.1177476 27121730

[53] Jayanthi N Pinkham C Dugas L Patrick B LaBella C. Sports specialization in young athletes:evidence-based recommendations. Sports Health. (2013) 5(3):251–7. 10.1177/1941738112464626 24427397 PMC3658407

[54] Moseid CH Myklebust G Fagerland MW Bahr R. The association between early specialization and performance level with injury and illness risk in youth elite athletes. Scand J Med Sci Sports. (2019) 29(3):460–8. 10.1111/sms.13338 30450649

[55] Côté J Vierimaa M. The developmental model of sport participation: 15 years after its first conceptualization. Sci Sports. (2014) 29:S63–S9. 10.1016/j.scispo.2014.08.133

[56] Gonzalez AM Hoffman JR Rogowski JP Burgos W Manalo E Weise K Performance changes in NBA basketball players vary in starters vs. Nonstarters over a competitive season. J Strength Cond Res. (2013) 27(3):611–5. 10.1519/JSC.0b013e31825dd2d9 22648143

[57] Caterisano A Patrick BT Edenfield WL Batson MJ. The effects of a basketball season on aerobic and strength parameters among college men: starters vs. Reserves. J Strength Cond Res. (1997) 11(1):21–4.

[58] Ferioli D Bosio A Zois J La Torre A Rampinini E. Seasonal changes in physical capacities of basketball players according to competitive levels and individual responses. PloS one. (2020) 15(3):e0230558. 10.1371/journal.pone.0230558 32191740 PMC7082009

[59] Gonzalez AM Hoffman JR Scallin-Perez JR Stout JR Fragala MS. Performance changes in national collegiate athletic association division I women basketball players during a competitive season: starters vs. Nonstarters. J Strength Cond Res. (2012) 26(12):3197–203. 10.1519/JSC.0b013e318273665d 22996019

[60] Guimarães E Baxter-Jones ADG Williams AM Tavares F Janeira MA Maia J. The role of growth, maturation and sporting environment on the development of performance and technical and tactical skills in youth basketball players: the INEX study. J Sports Sci. (2021) 39(9):979–91. 10.1080/02640414.2020.1853334 33225823

[61] te Wierike SC de Jong MC Tromp EJ Vuijk PJ Lemmink KA Malina RM Development of repeated sprint ability in talented youth basketball players. J Strength Cond Res. (2014) 28(4):928–34. 10.1097/JSC.0000000000000223 24667248

[62] McManus AM Armstrong N. Physiology of elite young female athletes. Med Sport Sci. (2011) 56:23–46. 10.1159/000320626 21178365

[63] Mengersen KL Drovandi CC Robert CP Pyne DB Gore CJ. Bayesian estimation of small effects in exercise and sports science. PloS one. (2016) 11(4):e0147311. 10.1371/journal.pone.0147311 27073897 PMC4830602

