# Supplementary material for: Does early specialization provide an advantage in physical fitness development in youth basketball?
Source: Front Sports Act Living. 2023 Jan 10;4:1042494. doi: 10.3389/fspor.2022.1042494 (PMC9872025; doi:10.3389/fspor.2022.1042494)
Supplement: Supplementary file 1 [file Datasheet1.pdf]

Supplementary material for the manuscript "Does early specialization provide an advantage in physical fitness development in youth basketball?"

André L A Soares[a], Ahlan B Lima[a], Caio G Miguel[a], Luciano Galvão[a], Thiago J Leonardi[b], Roberto R Paes[c],Carlos E Gonçalves[d], Humberto M Carvalho[a]

[a]Department of Physical Education, School of Sports, Federal University of Santa Catarina, Florianópolis, Santa Catarina, Brazil; [b]School of Physical Education, Physiotherapy and Dance, Federal University of Rio Grande do Sul, Porto Alegre, Rio Grande do Sul, Brazil; [c]Faculty of Physical Education, University of Campinas, Campinas, São Paulo, Brazil; [d]Faculty of Sport Sciences and Physical Education, University of Coimbra, Coimbra, Portugal

Supplementary Figures

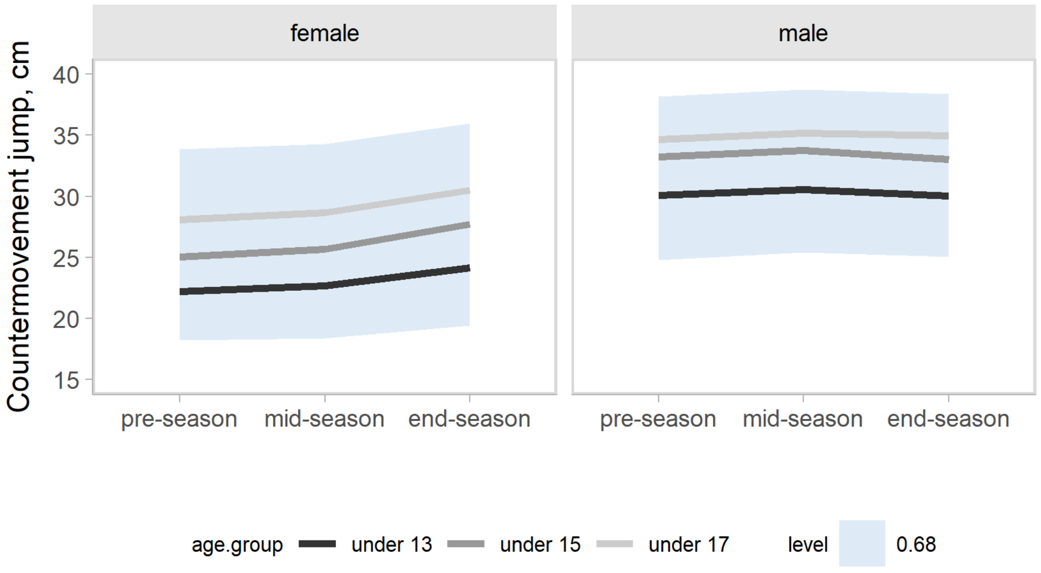

Supplementary Figure 1. Changes in countermovement jump performance for young females and male basketball players within a basketball season by age group. The shaded area represents the 68% credible interval, similar to a standard deviation.

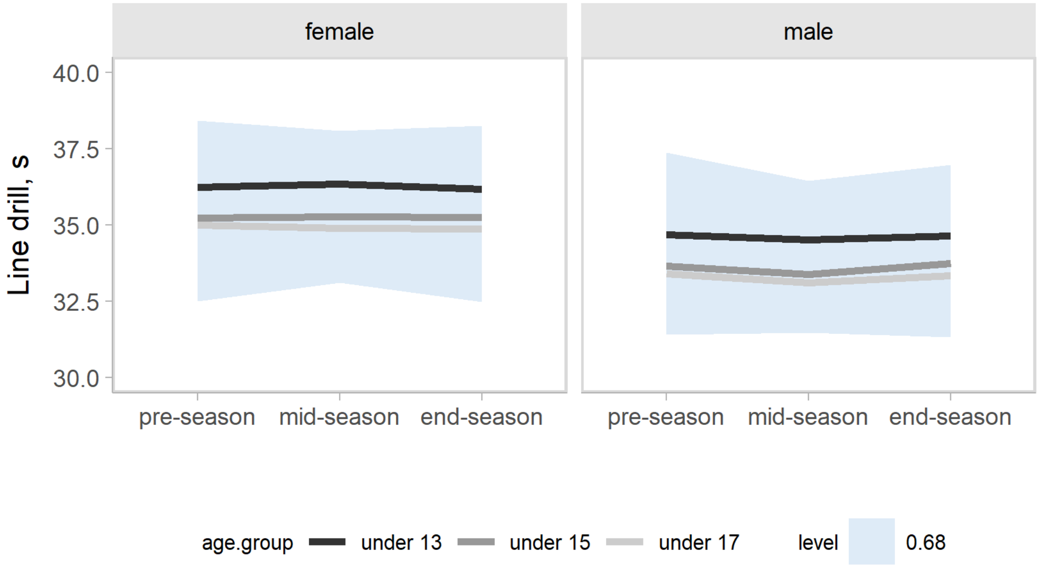

Supplementary Figure 2. Changes in line-drill performance for young females and male basketball players within a basketball season by age group. The shaded area represents the 68% credible interval, similar to a standard deviation.

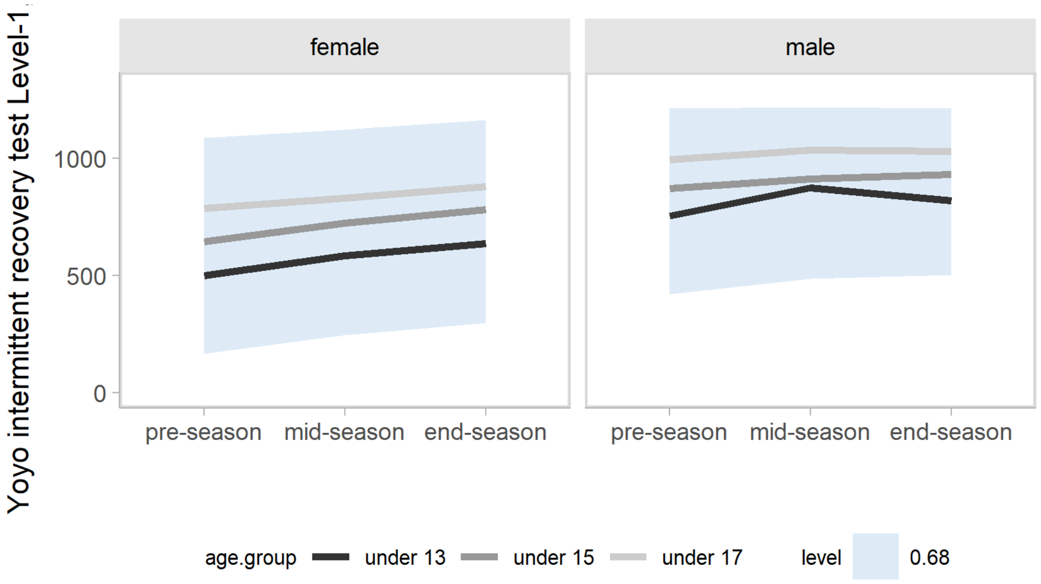

Supplementary Figure 3. Changes in Yo-yo intermittent recovery test level 1 performance for young females and male basketball players within a basketball season by age group. The shaded area represents the 68% credible interval, similar to a standard deviation.

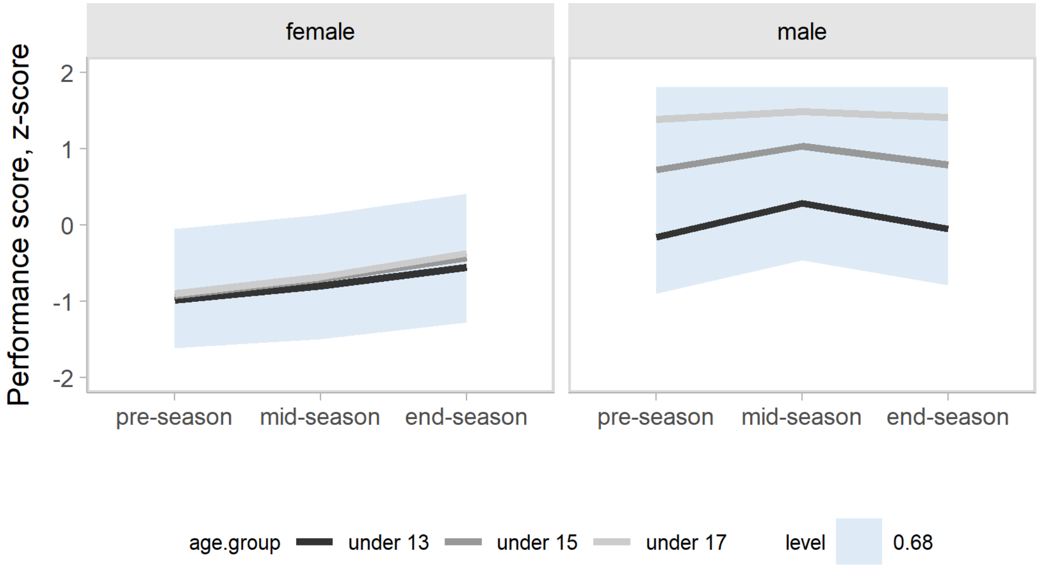

Supplementary Figure 4. Changes in overall basketball-specific physical fitness index for young females and male basketball players within a basketball season by somatic maturity status. The shaded area represents the 68% credible interval, similar to a standard deviation.

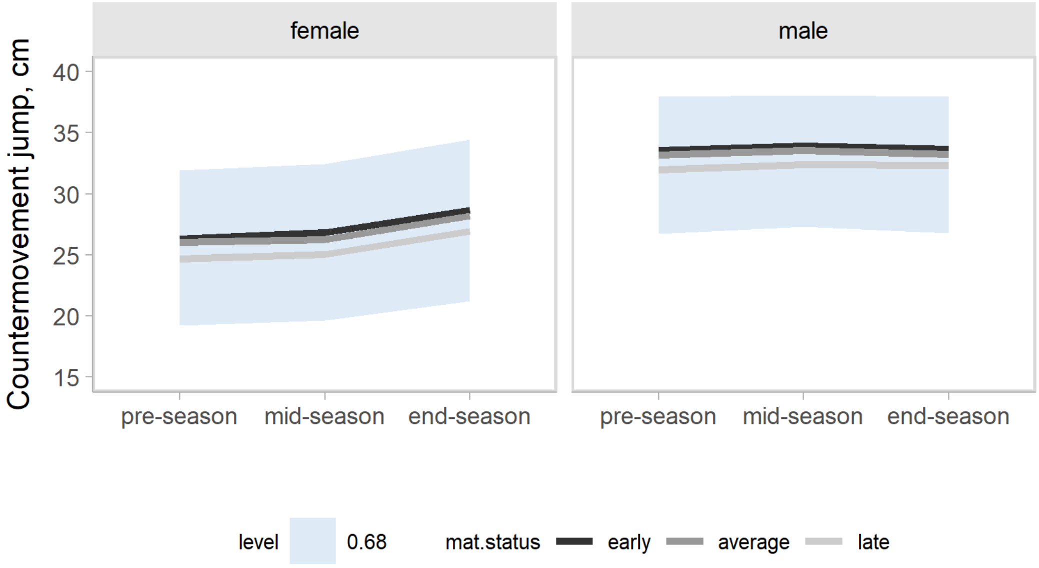

Supplementary Figure 5. Changes in Countermovement jump for young females and male basketball players within a basketball season by somatic maturity status. The shaded area represents the 68% credible interval, similar to a standard deviation.

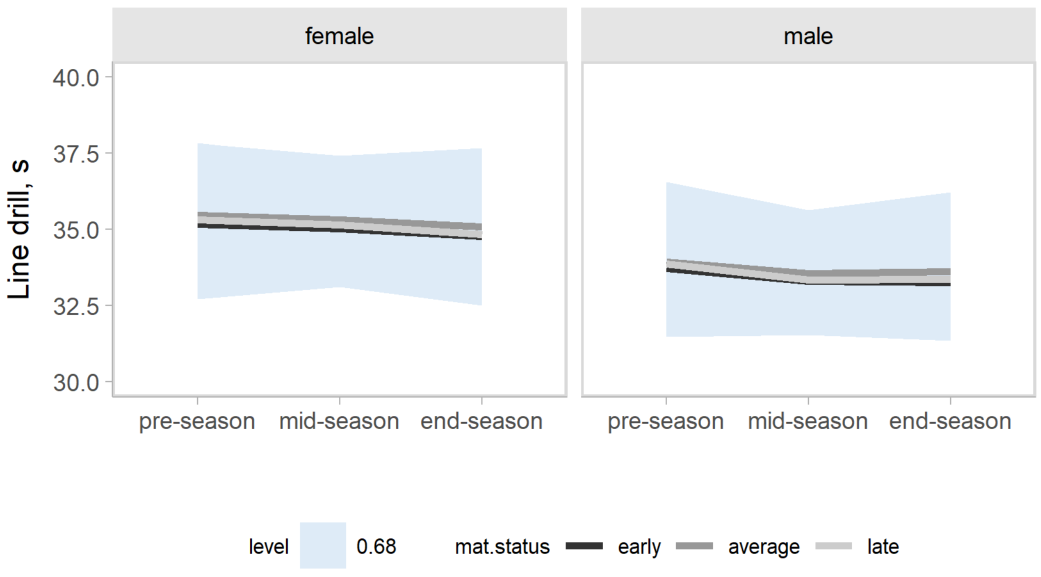

Supplementary Figure 6. Changes in line-drill performance for young females and male basketball players within a basketball season by somatic maturity status. The shaded area represents the 68% credible interval, similar to a standard deviation.

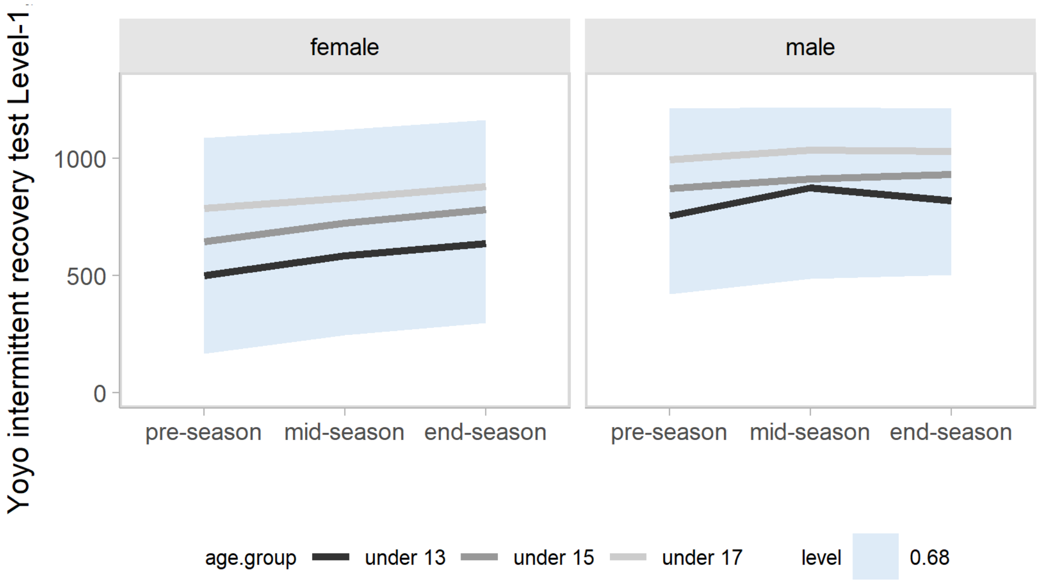

Supplementary Figure 7. Changes in Yo-yo intermittent recovery test level 1 performance for young females and male basketball players within a basketball season by somatic maturity status. The shaded area represents the 68% credible interval, similar to a standard deviation.

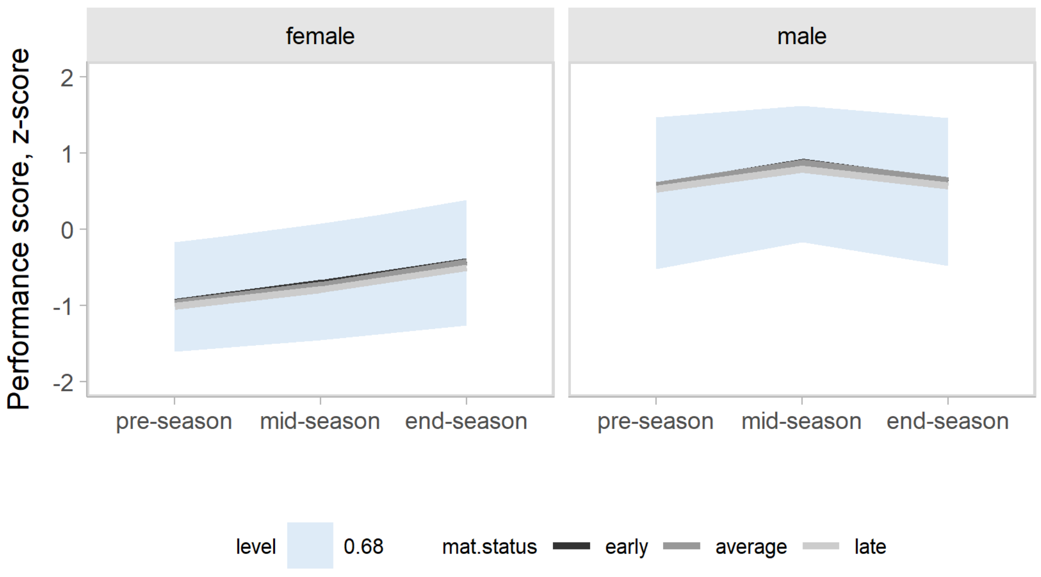

Supplementary Figure 8. Changes in overall basketball-specific physical fitness index for young females and male basketball players within a basketball season by somatic maturity status. The shaded area represents the 68% credible interval, similar to a standard deviation.

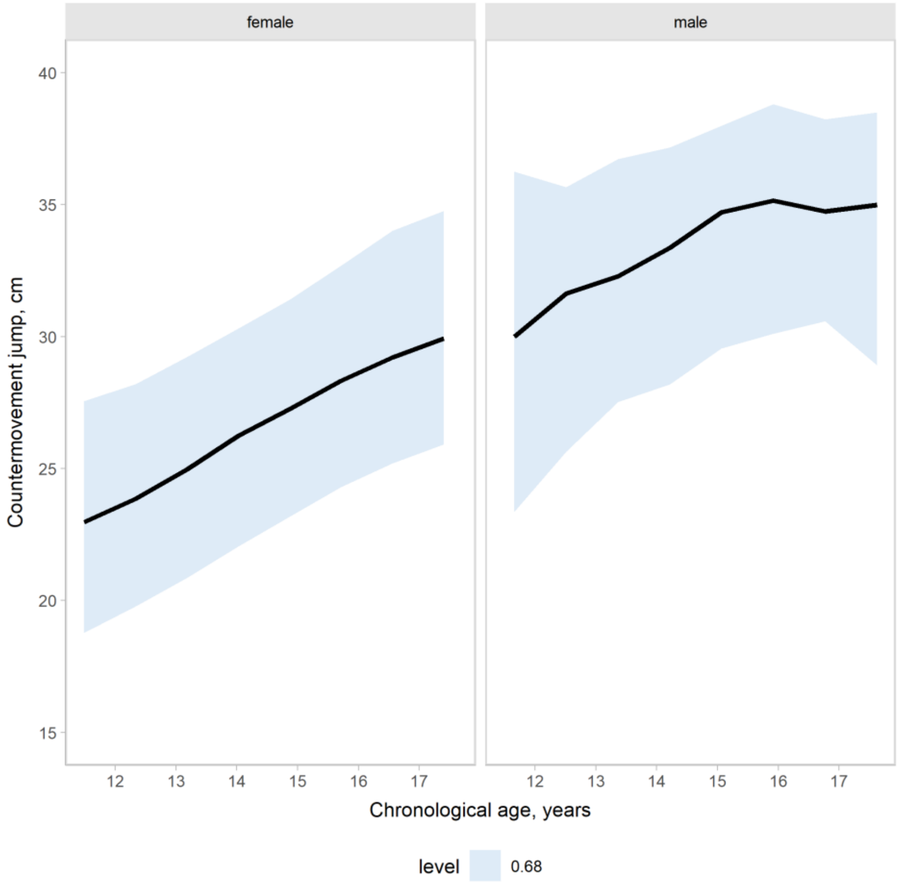

Supplementary Figure 9. Developmental changes in countermovement jump for young females and male basketball players within a basketball season. The shaded area represents the 68% credible interval, similar to a standard deviation.

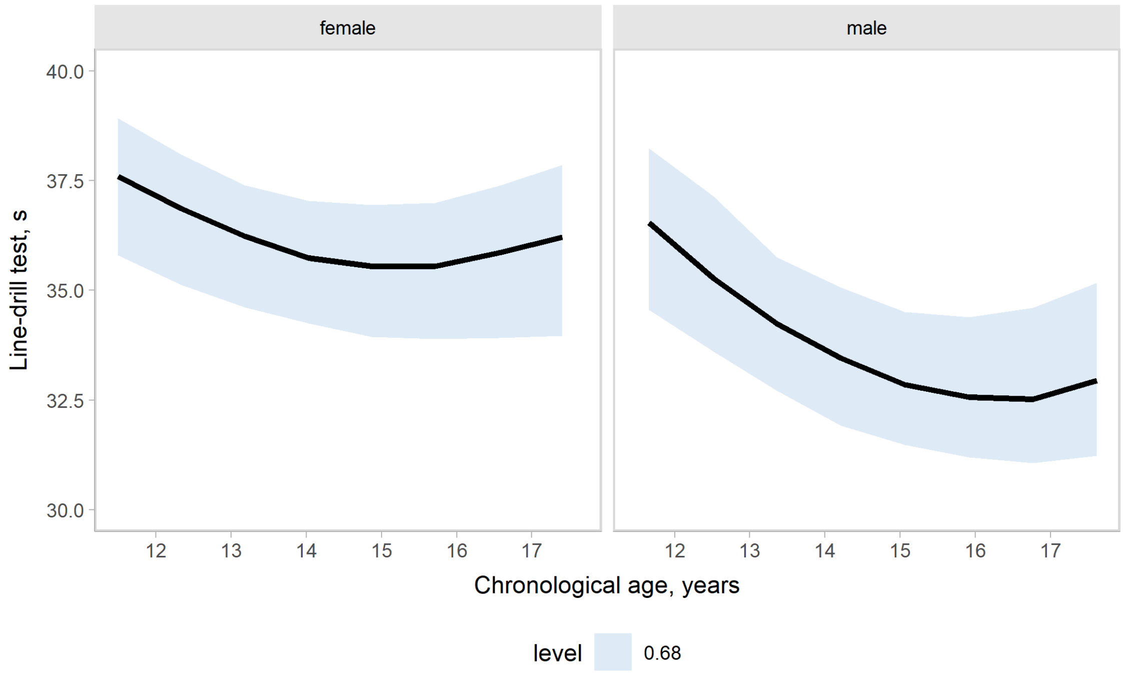

Supplementary Figure 10. Developmental changes in line-drill performance for young females and male basketball players within a basketball season. The shaded area represents the 68% credible interval, similar to a standard deviation.

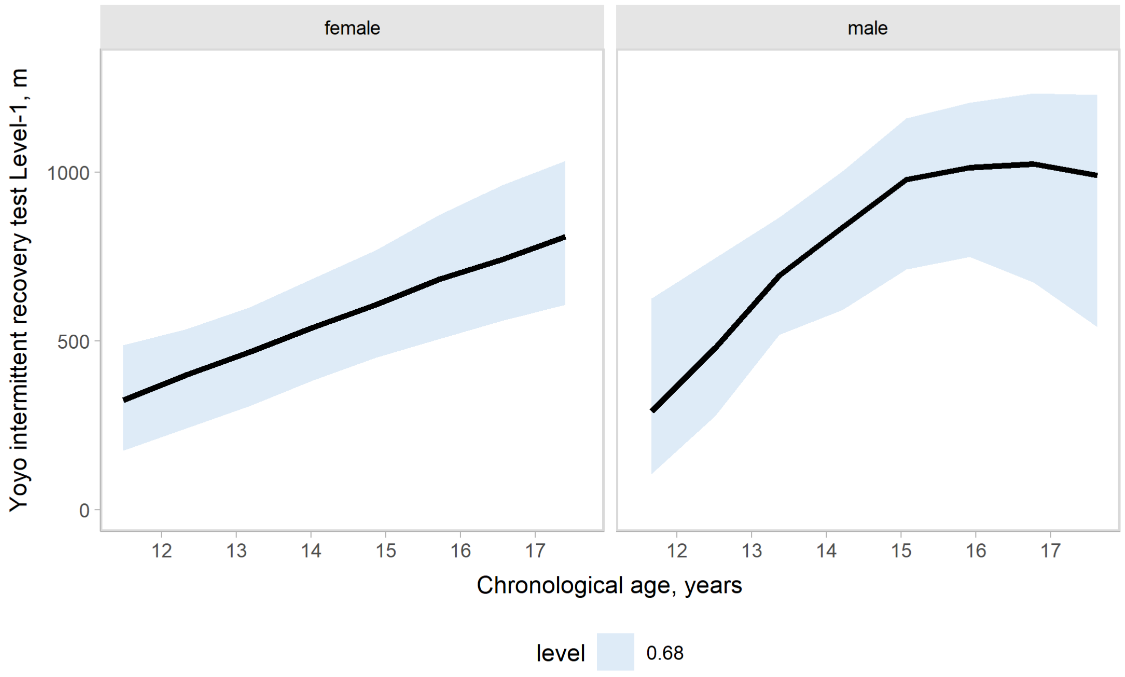

Supplementary Figure 11. Developmental changes in Yo-yo intermittent recovery test level 1 performance for young females and male basketball players within a basketball season. The shaded area represents the 68% credible interval, similar to a standard deviation.

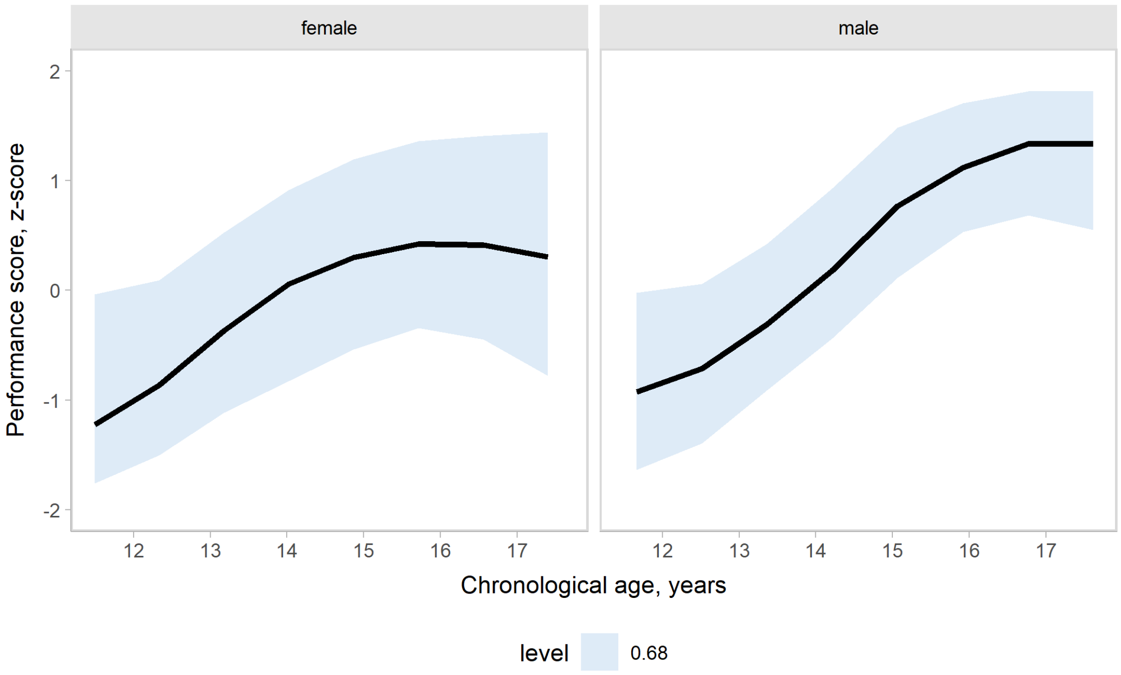

Supplementary Figure 12. Developmental changes in overall basketball-specific physical fitness index for young females and male basketball players within a basketball season. The shaded area represents the 68% credible interval, similar to a standard deviation.
